# HCV Elimination in Central Europe with Particular Emphasis on Microelimination in Prisons

**DOI:** 10.3390/v14030482

**Published:** 2022-02-26

**Authors:** Robert Flisiak, Dorota Zarębska-Michaluk, Egle Ciupkeviciene, Sylvia Drazilova, Sona Frankova, Ivica Grgurevic, Bela Hunyady, Peter Jarcuska, Limas Kupčinskas, Michael Makara, Gunita Saulite-Vanaga, Marieta Simonova, Jan Sperl, Ieva Tolmane, Adriana Vince

**Affiliations:** 1 Department of Infectious Diseases and Hepatology, Medical University of Białystok, 15-540 Białystok, Poland; 2 Department of Infectious Diseases, Jan Kochanowski University, 25-317 Kielce, Poland; dorota1010@tlen.pl; 3 Department of Gastroenterology, Lithuanian University of Health Sciences, LT50061 Kaunas, Lithuania; egle.ciupkeviciene@lsmuni.lt (E.C.); l.kupcinskas@gmail.com (L.K.); 4 2nd Department of Internal Medicine, L Pasteur University Hospital and PJ Safarik University, Faculty of Medicine, 04011 Kosice, Slovakia; drazilovasylvia@gmail.com (S.D.); peter.jarcuska@upjs.sk (P.J.); 5 Department of Hepatogastroenterology, Institute for Clinical and Experimental Medicine, 14021 Prague, Czech Republic; sona.frankova@ikem.cz (S.F.); jan.sperl@ikem.cz (J.S.); 6 Department of Gastroenterology, Hepatology and Clinical Nutrition, University of Zagreb, University Hospital Dubrava, 10000 Zagreb, Croatia; ivicag72@gmail.com; 7 Department of Gastroenterolgy, Somogy Megyei Kaposi Mór Oktató Kórház, H7400 Kaposvár, Hungary; bhunyady@yahoo.com; 8 First Department of Internal Medicine, University of Pécs, H7624 Pécs, Hungary; 9 Central Hospital of Southern Pest National Institute of Hematology and Infectious Diseases, Saint Laszlo Hospital, 5-7. Albert Florian Street, H1097 Budapest, Hungary; michael@makara.md; 10 Out-Patient Department, Riga East University Hospital, Latvian Center of Infectious Diseases, LV1013 Riga, Latvia; gunita.saulite@aslimnica.lv (G.S.-V.); ieva.tolmane@aslimnica.lv (I.T.); 11 Department of HPB Surgery and Transplantology, Clinic of Gastroenterology, Military Medical Academy, 1606 Sofia, Bulgaria; marietta.simonova@gmail.com; 12 Department of Infectious Diseases, Medical School University of Zagreb, University Hospital of Infectious Diseases, 10000 Zagreb, Croatia; avince@bfm.hr

**Keywords:** hepatitis, HCV, WHO, epidemiology, therapy, screening

## Abstract

In 2016, the WHO announced a plan to eliminate viral hepatitis as a public health threat by 2030. In this narrative review, experts from Bulgaria, Croatia, the Czech Republic, Hungary, Latvia, Lithuania, Poland and Slovakia assessed the feasibility of achieving the WHO 2030 target for HCV infections in Central Europe. They focused mainly on HCV micro-elimination in prisons, where the highest incidence of HCV infections is usually observed, and the impact of the COVID-19 pandemic on the detection and treatment of HCV infections. According to the presented estimates, almost 400,000 people remain infected with HCV in the analyzed countries. Interferon-free therapies are available ad libitum, but the number of patients treated annually in the last two years has halved compared to 2017–2019, mainly due to the COVID-19 pandemic. None of the countries analyzed had implemented a national HCV screening program or a prison screening program. The main reason is a lack of will at governmental and prison levels. None of the countries analyzed see any chance of meeting the WHO targets for removing viral hepatitis from the public threat list by 2030, unless barriers such as a lack of political will and a lack of screening programs are removed quickly.

## 1. Introduction

Direct-acting antivirals (DAA) have significantly improved the effectiveness and safety of Hepatitis C virus (HCV) infection treatment. This therapeutic revolution opened up the possibility of the effective treatment of almost all HCV-infected individuals. This has resulted in the commencement of research worldwide on the incidence of HCV infections and on the possibilities of its complete elimination as a public health problem [1,2,3]. Although new therapeutic regimens have eliminated the waiting lists for the treatment of diagnosed patients, a large group of patients with HCV remained undiagnosed and at risk of developing cirrhosis and/or hepatocellular carcinoma [3,4]. In 2016, the WHO announced a plan to eliminate viral hepatitis as a public health threat by 2030 [5]. However, the condition for achieving this goal is not only access to highly effective drugs, but also the ability to detect hidden infections by launching national screening programs. Due to the general lack of political will, despite proven cost-effectiveness, this goal may be difficult to achieve [6,7]. An additional difficulty in the elimination of HCV infections has been caused by the COVID-19 pandemic [8]. Although most of the analyzed countries belong to the group of high-income countries, according to the recent publication of the Center for Disease Analysis, none of them will achieve the goals set by the WHO [9].

In this narrative review, a team of experts from Bulgaria, Croatia, Czech Republic, Hungary, Latvia, Lithuania, Poland, and Slovakia assessed the current epidemiological situation in the field of HCV and the possibility of achieving the goals set by the WHO. The effect of the pandemic on the detection and treatment of HCV infections in Central Europe is the additional subject of this article. We paid special attention to the micro-elimination of HCV infections in prisons, where the highest incidence of infections is usually observed.

## 2. Methodology

Knowledge about the current epidemiological situation, diagnostic and therapeutic possibilities and actions taken in the vast majority of countries is available at best in the form of reports and publications in local languages. Therefore, the only way to analyze this type of data is to use the knowledge of experts from individual countries. To evaluate these data, a modified Delphi process was adopted that used multiple rounds of structured feedback to reach consensus on key points of interest and end comments. The data collection process began with the invitation of experts based on their previous involvement in the activities of the Central European Hepatologic Collaboration, which is the initiator of this study. Additionally, representatives of hepatological or gastroenterological societies from other countries included in Central Europe as broadly understood were invited. Experts from eight countries—Bulgaria, Croatia, the Czech Republic, Latvia, Lithuania, Slovakia, Poland and Hungary—have declared their readiness to participate in the development of the data. Following a virtual meeting on 19 October 2021, a survey describing topics of interest was sent by e-mail to experts. The questionnaire focused on collecting national data grouped into six main themes: epidemiological situation, treatment options, impact of COVID-19 on access to treatment, national screening program, prison screening program, and the possibility of achieving the WHO 2030 target. Each topic included questions with a choice of answers and an additional option to describe the situation in a given country. Responses to the questionnaire were collected and analyzed, and then sent to all project participants by e-mail on 11 November 2021 in the form of a draft report with tables and a descriptive section dedicated to each country. After receiving the feedback, it was necessary to repeat the e-mail exchange of the questionnaires twice in order to obtain the final shape of the report and the article. Consensus was reached when all participants had no further substantive comments and approved the final version for publication.

## 3. Epidemiologic Situation

**Figure 1:**
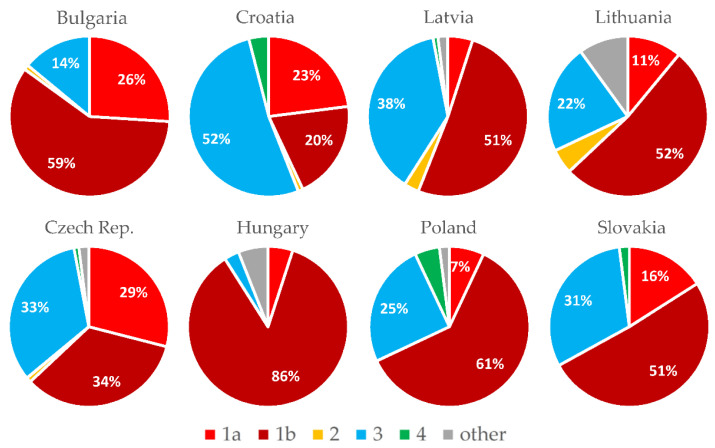
Genotype prevalence in 2020/2021; “other” are mixed genotypes or genotype 1 without specifying a subtype.

The current prevalence rates and estimated number of HCV infected in particular countries are presented in Table 1, whereas the prevalence of genotypes is illustrated in Figure 1.

### 3.1. Bulgaria

The prevalence of HCV infection was estimated at 1.28% in a multicenter study from 2000, which means 80,000 viremic individuals. The incidence of HCV has been stable over the past 15 years, and in 2015 it was 1.18 per 100,000. As shown in Figure 1, the dominant genotype (G) of HCV in Bulgaria is G1b, found in 59% of those infected [10]. People who inject drugs (PWID) represent the main HCV risk population in Bulgaria, with a steadily increasing incidence of HCV, which was 76.8% in 2017 [11]. High HCV seroprevalence is also noted among prisoners (26%), the Roma community (24.6%) and sex workers (11%) [12,13,14].

### 3.2. Croatia

The estimated prevalence of HCV RNA is 0.6%, i.e., 20,000 inhabitants, and approximately 50% have been diagnosed, of which 55% have already been cured. Most of the newly diagnosed patients are PWID, and the prevalence of anti-HCV in this population is 34.7%, according to the 2019 data [15]. The most common genotype in 2020 was G3a (52%) (Figure 1). As of 2018, approximately 30% of newly diagnosed patients had fibrosis grade 4 (F4), as determined by transient elastography [16]. In 2020, there were only 90 newly reported cases of HCV, compared to an average of 200 cases before 2020. Screening is sporadic in the general population and in risk groups [17].

### 3.3. Czech Republic 

This is a country with a low HCV prevalence (0.5%), which is a little bit higher among adults with risk factors (0.8%). Most new cases of HCV are diagnosed among PWID (75%) and referred for treatment by harm reduction services and psychiatric hospitals [18,19]. The genotype distribution remains stable (Figure 1), but in the group of patients without a history of intravenous use of drugs, G1b still dominates (62%). A constant percentage of approximately 15–20% of newly diagnosed cases have cirrhosis [20]. The number of new HCV cases diagnosed in 2020 dropped to 770 due to the weakening of screening programs during the COVID-19 pandemic [21]. HCV patients are treated in 23 dedicated centers.

### 3.4. Hungary

Based on unrepresentative and usually outdated epidemiological studies conducted in the past, the incidence of HCV infection in Hungary is low [22]. The estimated HCV RNA positivity is 0.3% (approximately 30,000 people). The estimated incidence is 15/100,000 per year, with approximately 1500 new infections per year. However, the current diagnosis rate is only around 500 per year due to the low screening activity in part due to the COVID-19 pandemic. Persistently high HCV seropositivity was confirmed in the PWID population (40–50%) and in prisons (8–12%) [23]. G1a and G3 are the dominant genotypes in these high-risk groups, shifting the genotype distribution in these directions from traditional G1b dominance in the entire infected population [24].

### 3.5. Latvia

In Latvia, the incidence of HCV infection is relatively high. The prevalence of anti-HCV is 2.4% and the frequency of HCV RNA is 1.7%. However, in recent years, there has been a decrease in newly diagnosed cases. It is difficult to distinguish whether this reduction is due to effective treatment and an actual decrease in the number of infected patients, or the COVID-19 pandemic limiting the detection of infections [25]. The distribution of genotypes remains stable (Figure 1).

### 3.6. Lithuania

According to estimates based on research published in 2015 [26], the overall prevalence of anti-HCV antibodies in Lithuania was 1.7%, and the HCV viral load was estimated at 1.1%. Since 2019, an anti-HCV seroprevalence study has been underway in three regions of Lithuania, with 25,000 adults under the care of general practitioners. This study demonstrated a lower seroprevalence of 1.4% and a viral prevalence of 0.9%. [27]. According to the official Lithuanian registry of acute infections, the number of acute hepatitis C cases has also gradually decreased from 2.0 per 100.000 inhabitants in 2005 to 0.9 per 100.000 in 2018 [28]. Genotype 1b is still the most common genotype in Lithuania among patients with hepatitis C. [29].

### 3.7. Poland

The prevalence of active infection with HCV is estimated at about 0.4% of the population, i.e., about 140,000 inhabitants. The vast majority of these have not been diagnosed yet [30]. The situation is not aided by the fact that high-risk groups are not screened. As shown in Figure 1, G1b is dominating, followed by G3 and G4, and infections with other genotypes have been practically absent in Poland in recent years. Compared to previous years, the percentage of those infected with G3 has increased and G1b has decreased. As of 2018, a percentage of approximately 20% of those newly diagnosed and qualified for treatment have been diagnosed with cirrhosis of the liver [31,32].

### 3.8. Slovakia

The prevalence of chronic hepatitis C with HCV RNA in Slovakia is 0.2%, which means that 10,000 people are infected. About half of the infected patients are assumed to be PWID and prisoners [33]. The distribution of hepatitis C genotypes in Slovakia is shown in Figure 1 [34].

## 4. Treatment Opportunities

**Figure 2:**
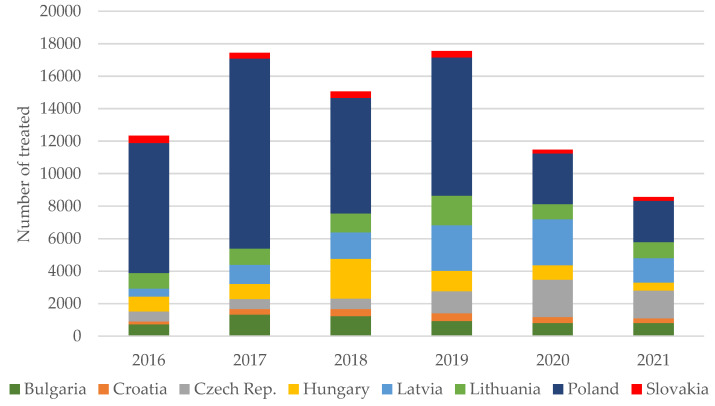
Proportions of patients treated in 2016–2021 by country.

**Figure 3:**
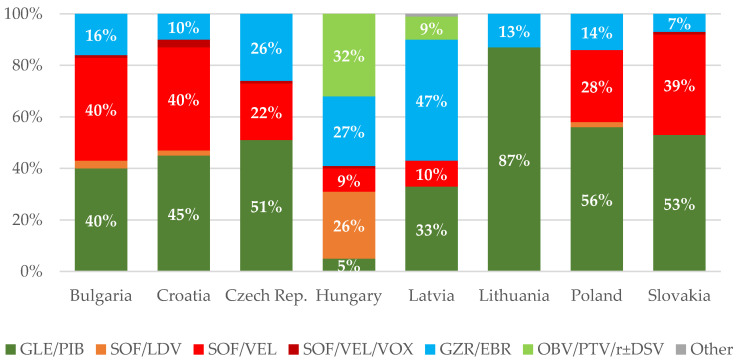
Proportions of regimens administered in 2020/2021 by country; glecaprevir/pibrentasvir (GLE/PIB), sofosbuvir/ledipasvir (SOF/LDV), SOF/velpatasvir (SOF/VEL), SOF/VEL/voxilaprevir (SOF/VEL/VOX), grazoprevir/elbasvir (GZR/EBR), ombitasvir/paritaprevir/ritonavir ± dasabuvir (OBV/PTV/r ± DSV).

The numbers of patients treated for HCV between 2016 and 2021 are provided in Table 1. In addition, the proportions of treated patients and regimens administered to patients in particular countries are illustrated in Figure 2 and Figure 3, respectively.

### 4.1. Bulgaria

The following treatment regimens are available and fully reimbursed by the National Health Insurance Fund (NHIF) in Bulgaria: glecaprevir/pibrentasvir (GLE/PIB), sofosbuvir/ledipasvir (SOF/LDV), SOF/velpatasvir (SOF/VEL), SOF/VEL/voxilaprevir (SOF/VEL/VOX), and grazoprevir/elbasvir (GZR/EBR). DAA antiviral treatment has been reimbursed in Bulgaria since 2016, and all restrictions on treating patients with health insurance were removed in 2017. However, around 12% of the country’s total population and more than 50% of marginalized populations, such as PWID, have no health insurance [11]. Therapy can only be ordered at Gastroenterology Clinics nominated by the National Health Fund.

### 4.2. Croatia

Treatment is 100% reimbursed and all EU-registered interferon-free options are available. There are no restrictions on access to treatment depending on the severity of the disease, with the exception of PWID, where a 6-month abstinence from injecting illegal drugs is required by the National Health Insurance Fund. The combination of SOV/VEL has been approved for children 6 years of age and older from 2021. The SOF/VEL/VOX combination is only recommended for patients who have failed the first line of oral antiviral therapy.

### 4.3. Czech Republic

GLE/PIB, SOF/VEL and GZR/EBR are reimbursed for the first-line treatment, whereas SOF/VEL/VOX is approved for previous DAA failures. No restriction criteria are applied for treatment initiation, and all HCV-infected patients have access to therapy.

### 4.4. Hungary

All interferon-free therapies are available, except GZR/EBR, which was recently phased out in Hungary [35]. The addition of ribavirin (RBV) is allowed only when a combination without RBV is not available or potentially ineffective in the opinion of the treating physician (mainly decompensated cirrhosis or retreatment). Due to previous contracts of the National Health Fund, non-pangenotypic combinations were still used in patients with G1b infection in the years 2020–2021. Patients with G3 and G1a infection (especially after GZR/EBR failure) are treated with SOF/VEL or GLE/PIB. SOF/VEL/VOX is only available on a case-by-case basis for patients who have failed a previous DAA regimen containing NS5A.

### 4.5. Latvia

The treatment of HCV infection is 100% reimbursed, and there are no restrictions, but qualification for treatment requires consultation with an infectiologist. Treatment is allowed in five centers in Latvia. GZR/EBR for G1 and G4 and GLE/PIB, SOF/VEL for G2 and G3 are reimbursed. In the case of treatment failure with G1 and G4, approval should be requested for the use of pangenotypic regimens [36,37]. Reimbursement of SOF/VEL/VOX therapy is expected in 2022.

### 4.6. Lithuania

Two IFN-free therapies with GLE/PIB and GZR/EBR are reimbursed by the National Health Insurance Fund. Sofosbuvir-based therapies have been used only in a few patients (10 cases, 0.5%) after failure of the above-mentioned therapies or after liver transplantation.

### 4.7. Poland

All EU-registered interferon-free options are available, but in practice, 84% are pangenotypic therapies with GLE/PIB or SOF/VEL. Rescue therapy with SOF/VEL/VOX is being reimbursed from 2021. There is no restriction in access to drugs depending on the severity of the disease or other factors [32,38].

### 4.8. Slovakia

GLE/PIB, SOF/VEL, SOF/VEL/VOX and GZR/EBR are being reimbursed by health insurance companies in Slovakia from 2021. In the event of the failure of the first-line treatment, re-treatment reimbursement is considered individually. There are serious barriers to the treatment of prisoners and PWID in Slovakia [39].

## 5. Effect of COVID-19 on Access to Treatment

The effect of the COVID-19 pandemic on the elimination of HCV in particular countries is summarized in Table 2.

### 5.1. Bulgaria

The COVID-19 pandemic has reduced access to HCV testing and treatment. The activities of gastroenterology departments, which are the main centers for HCV infection diagnosis and DAA therapy, were limited in 2019–2020, when some of them were converted to COVID-19 treatment units. Low rates of screening and diagnostic tests outside hospitals were also strong limiting factors. The number of patients treated in 2021 was approximately 800, which is a decrease compared to the years preceding COVID-19.

### 5.2. Croatia

Treatment is available, but diagnostic options are reduced as GPs and specialists mainly work with COVID-19, which has resulted in fewer patients being referred for specialist evaluation, thus worsening the linkage to care.

### 5.3. Czech Republic

The number of treated patients decreased owing to the COVID-19 pandemic in 2021 [21,40]. This decrease is multifactorial: lower numbers of screened and diagnosed patients, especially those at risk of harm; the reduction in services; the lower number of patients starting treatment at HCV centers, which have been overtaken by infectious disease specialists who are taking care of COVID patients and organizing vaccination programs.

### 5.4. Hungary

The rates of screening, diagnosis and treatment fell drastically due to the diminished screening activity in 2020–2021, partly due to the lack of an effective national screening program, itself partly due to the COVID-19 pandemic. Due to the pandemic, diagnostic and treatment options have also decreased, and a significant number of professionals (doctors and nurses) have been directed to COVID-19-related services. The number of newly diagnosed patients entering treatment decreased from 1.267 in 2019 to 896 in 2020, and is expected to be below 500 in 2021.

### 5.5. Latvia

Access to treatment is limited due to the COVID-19 pandemic and the resulting difficulty of access to infectiologists, as well as the reduced number of outpatient consultations, limited to acute conditions.

### 5.6. Lithuania

The impact of COVID-19 on the number of hepatitis C patients treated in Lithuania has been significant. In 2020, only 51% of patients with hepatitis C were treated, compared to those treated in 2019. The number of treated patients in 2021 was similar to that in 2020 (Table 1).

### 5.7. Poland

COVID-19 has stopped almost all screening activities. The exceptions are in the screening of people vaccinated against COVID-19 in some centers. Due to the fact that almost all centers treating HCV infections are infectious disease centers, which have been almost entirely dedicated to COVID-19, access to therapy, even for those diagnosed, has been difficult and sometimes impossible. In addition, this has been affected by the fear patients have of reporting to health care facilities. As a result, in 2020, the number of patients treated decreased by 63% compared to 2019, and a further reduction is expected in 2021.

### 5.8. Slovakia

The COVID-19 pandemic has delayed HCV elimination in Slovakia, because access to hepatology and infectious diseases clinics is limited. Infectiologists mainly focus on COVID-19 infection. This has led to a deterioration in the diagnosis and treatment of chronic hepatitis C. In 2020, a 40% reduction in the number of patients treated with HCV was observed compared to the previous year. We do not expect the number of patients treated in 2021 to increase.

## 6. National Screening Program

The availability of national screening programs and the reasons for not having them are presented in Table 3.

### 6.1. Bulgaria

A national eradication plan for viral hepatitis was approved in 2021, but this has not been actively implemented, and funding has not been provided due to a lack of understanding and political will. The national screening program is expected to target people aged 40 to 65. Screening will also be carried out in risk groups such as prisoners, men who have sex with men (MSM), sex workers, ethnic minorities, and migrants.

### 6.2. Croatia

The national action plan to prevent viral hepatitis was fully written out in 2019 and presented to the former Minister of Health, who endorsed it and sent it for further stakeholder consultation before being approved by the government. Unfortunately, the COVID-19 pandemic has interrupted this project, and a plan to combat viral hepatitis is not currently in the spotlight of the health administration.

### 6.3. Czech Republic

National screening program guidelines have been developed and are undergoing a scientific and cost assessment by the Ministry of Health and by teams of experts in various fields involved in screening strategies (gynecologists, diabetologists, prison services). So far, no national screening has been launched, but it is planned for 2022. The delay is due to the COVID-19 pandemic.

### 6.4. Hungary

In Hungary, it has not been developed and is showing slow progress in preparation. COVID-19 has had a negative impact on the activities of the authorities and doctors involved. Recent government action (from 2019) has included mandatory screening for health professionals [41].

### 6.5. Latvia

The only national HCV screening program is for blood donors. Doctors also screen pregnant women, individuals on dialysis and HIV-infected patients. Examination is sometimes offered to inmates, but it depends on the prison. In 2021, at the initiative of infectiologists, screening tests for people staying in shelters, psychoneurological hospitals and social welfare centers were started. The preliminary results show a higher prevalence of anti-HCV antibodies in these three groups compared to the general population. Screening for the whole population is not considered to be cost-effective due to the predicted low prevalence (similar explanations given for national breast and colorectal cancer screening). It is believed that a more effective method may be to increase awareness and possible screening in groups at risk of HCV infection.

### 6.6. Lithuania

The full national screening program in Lithuania is still under preparation, and has not been finalized. However, in December 2021, the Lithuanian health authorities agreed, as a first step in the program, to pay GPs a “special fee for a promotional service” to perform serological tests for HCV on the population born 1945–1994. The project will start on 1 January 2022. Annual HCV testing by GPs is also planned for PWID and AIDS patients.

### 6.7. Poland

The only currently performed screening tests at the national level concern blood donors and those infected with HIV. For many years, numerous projects have been considered, and have not been implemented on a massive scale, with the exception of small pilot actions at most [30]. There is currently no political will to act on screening for HCV despite promises from time to time. 

### 6.8. Slovakia

There is no national HCV screening program in Slovakia due to the lack of political will and insufficient financing. The low prevalence of HCV infection in the general population is another reason why a national screening program is not planned.

## 7. Screening Program in Prison

The availability of national screening programs in prisons and the reasons for not having them are presented in Table 4.

### 7.1. Bulgaria

To date, no national prison screening program has been implemented. Previously published data show an HCV seroprevalence of 14% and 26% in 2006 and 2011, respectively [14]. In 2020–2021, activities in the field of the screening and treatment of HCV-infected prisoners were initiated by non-governmental organizations and the nationwide gastroenterology association. They were implemented with the support of pharmaceutical companies. Such action has been taken in nine out of twelve prisons in Bulgaria, but no results have been presented so far.

### 7.2. Croatia

The prevalence of HCV among prisoners is estimated at 8.3 to 44% [42]. So far, there are no screening programs in prisons, except for occasional screenings at the request of inmates themselves. Treatment of inmates is 100% reimbursed by the National Health Insurance Fund. Prison medical staff is scarce, and prison officials say they are not adequately equipped to undergo screening tests and supervise treatment. However, the central prison authorities of the Croatian Ministry of Justice are interested in improving screening and treatment, and the project, developed in collaboration with an NGO, started in September 2021 [43]. The project covers three main prisons where inmates will be offered testing, further diagnosis and treatment on a voluntary basis. The project will be supported by doctors and nurses from hospitals.

### 7.3. Czech Republic

Prison screening is carried out regularly in the Czech Republic (in line with the medical guidelines of the Prison Service). All people arrested or imprisoned are tested for blood-borne infections and sexually transmitted diseases, which include HCV infection, which is found in 62.6% of respondents. About 90% of diagnosed patients start treatment in a prison. Treatment of HCV infected patients is not provided only for short-term detentions.

### 7.4. Hungary

Previous screenings (25,000 prisoners over 10 years) and treatments (>1000 prisoners over 10 years) were halted due to the COVID-19 pandemic and changes in sponsorship. The existing program has not been funded by the government, but it is expected to become involved from 2022 (program under preparation).

### 7.5. Latvia

There is no national screening program in prisons, but people in prison are offered blood screening tests (primarily for HIV), and testing is not compulsory. There are nine prisons in Latvia, and the percentage of people tested for HCV ranges from 30 to 50%, although in one of these prisons it reaches 100% (the adviser is an infectiologist). The reason for this relatively low testing rate is that not all inmates are offered screening for HCV. About 90% of HCV infected people diagnosed in prisons receive treatment.

### 7.6. Lithuania

According to data from 2020, there are 6751 prisoners in Lithuania. In 2018, a study on imprisonment rates in 28 EU countries showed that Lithuania has the highest, at 235 inmates per 100,000 inhabitants. There is no specific HCV screening program in prisons. Inmates diagnosed with hepatitis C, like other residents of the country, may receive DAA treatment reimbursed by the National Health Insurance Fund at the Ministry of Health. There is no needle and syringe exchange program in prisons in Lithuania [44].

### 7.7. Poland

There is no program of testing prisoners, but as a result of antibody testing in previous years, no more than 7% of prisoners have been tested. The reason is ignorance of the problem at the level of the central prison authority and the reluctance of the staff. Treatment is financed by a separate, very limited budget, and as a result very few HCV-infected patients are treated in prisons. In 2021, the Ministry of Health launched a discussion on screening and treatment in prisons, but action was hampered by another wave of COVID-19. Preparatory work has started recently, but implementation of the program is still uncertain.

### 7.8. Slovakia

A screening program for Slovak prisons is under preparation. Prisoners who are at risk of transmitting HCV infection are tested for HCV infection. About 22% of all inmates are tested for HCV infection every year. The anti-HCV positivity in this group of prisoners is 14%, which is many times more than in the general population. About 60% of HCV-infected prisoners are PWID. Approximately 11% of prisoners infected with HCV begin treatment while in prison. The lack of health insurance and the use of intravenous drugs are the main barriers in the treatment of chronic hepatitis C in this group of patients.

## 8. Is WHO 2030 Target Possible to Achieve in Your Country?

### 8.1. Bulgaria

The elimination goals of WHO 2030 are impossible to achieve with current diagnostic and therapeutic indicators. Another important obstacle to the eradication process is the lack of appropriate harm reduction measures to prevent new infections and the spread of infections in high-risk populations.

### 8.2. Croatia

There is still a chance to meet the WHO target, as all diagnosed patients are treated, but the main problem remains the lack of screening, which can lead to the persistence of a significant number of unrecognized and untreated [45] infections.

### 8.3. Czech Republic

The WHO target is not achievable owing to a low number of treated patients [18].

### 8.4. Hungary

The goals set by WHO are impossible to achieve in Hungary by 2030.

### 8.5. Latvia

Before the COVID-19 pandemic, it was assumed that the WHO target could be achieved in Latvia, but the situation has changed in the last two years. There has been a significant decrease in the number of patients qualified for treatment and treated for HCV. Therefore, it is likely that Latvia will not be able to achieve the goals set by the WHO by 2030.

### 8.6. Lithuania

Infection with COVID 19 has caused the discontinuation of preparations for the national HCV screening program. However, the decision to refund EUR 14.3 to family doctors for each HCV serological test performed in the population born in 1945–1994 raises faith in the achievement of the WHO 2030 target in Lithuania. However, an organizational effort will be required to encourage and motivate GPs to screen and refer seropositive patients to specialists (gastroenterologist or infectiologist) for treatment.

### 8.7. Poland

According to estimates, theoretically, the goal from 2022 is for 3 million citizens to be tested annually, which will allow 13,000 patients to be treated every year. In practice, due to the lack of political will, this goal is unattainable [31].

### 8.8. Slovakia

If the barriers to the treatment of chronic hepatitis C in PWID are maintained, it will be impossible to achieve HCV elimination by 2030 in Slovakia in line with the goals set by the WHO. Only the rapid removal of barriers to the treatment of PWID will help achieve this goal.

## 9. General Overview

**Figure 4:**
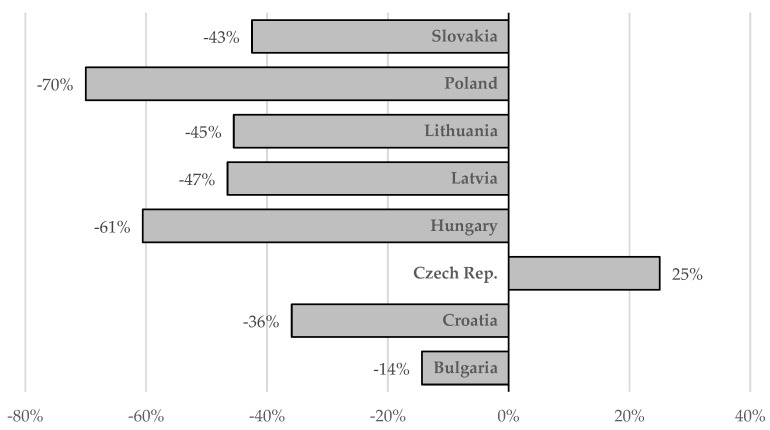
Change in the number of patients treated in 2021 compared to 2019 in individual countries.

According to the presented estimates, almost 400,000 people remain infected with HCV in the eight analyzed countries of Central Europe. The highest incidence is observed in Latvia, Bulgaria and Lithuania, and the lowest in Slovakia, Hungary and Poland (Table 1). A large majority of patients are infected with genotype 1 or 3, very few with 2 or 4, and there are no genotype 5 or 6 cases. Subgenotype 1b remains the dominant one, with the exception of Croatia, where genotype 3 is predominant (Figure 1). In most countries of the region, new infections are most often identified among PWID and prisoners. Interferon-free therapies are available without restrictions in all analyzed countries (Figure 3), but the number of patients treated annually in the last two years has decreased by half compared to 2017–2019 (Figure 2). However, a comparison of the situation in this respect between 2019 and 2021 in individual countries shows significant differences, from a reduction by 70% in Poland to an increase by 25% in the Czech Republic, which was the only country where there was an increase in the number of patients treated (Figure 4). The main reason for such a drastic reduction in the number of patients treated for HCV infection is the COVID-19 pandemic, which has reduced screening capacity, impeded access to health care, and led to a shift of medical personnel to care for patients with COVID-19 (Table 2). Interferon based therapies are no longer used in any country. In all but Hungary and Latvia, pangenotypic therapies were dominant, with GLE/PIB being the most common and VOX/VEL/SOF arising sporadically (Figure 3).

None of the analyzed countries implemented a national HCV screening program before or during the pandemic. In some countries, such programs have been under preparation for many years, but implementation is hampered by a lack of political will and insufficient financial resources (Table 3). Additionally, no country has implemented a prison screening program, despite prisons being the places with potentially the highest incidence of HCV infection. The main reason for this is a lack of will at the government and prison levels and, consequently, insufficient funding and medical staff (Table 4). In Bulgaria, Czech Republic and Hungary, almost all prisoners are offered screening tests despite the lack of national screening programs. In the Czech Republic, Hungary, Latvia and Lithuania, the vast majority of those diagnosed as infected with HCV have a chance to receive treatment during imprisonment. It seems possible to launch a screening program in the near future in Croatia. Very preliminary steps in this direction have been taken in Poland. However, in all countries there are still severe restrictions on access to screening and treatment for prisoners.

None of the countries analyzed see any chance of meeting the WHO targets for removing viral hepatitis from the list of public threats by 2030, unless barriers such as lack of political will and lack of screening programs are removed in the near future, and, in the case of Slovakia, restrictions of access to treatment for PWID are removed too. There is some hope that the WHO goals will be achieved in Lithuania by introducing screening reimbursement for GPs, but this will still require considerable effort. Unfortunately, the COVID-19 pandemic has significantly reduced the chance of achieving the goals set by the WHO.

## Figures and Tables

**Table 1:** Current HCV RNA prevalence and number of those treated in years 2016–2021 in selected Central European countries according to data provided by national experts.

	Bulgaria	Croatia	Czech Rep.	Hungary	Latvia	Lithuania	Poland	Slovakia
HCV RNA (+) prevalence-n, %	80,000	20,000	40,000	30,000	40,000	25,500	140,000	10,000
	1.1%	0.6%	0.5%	0.3%	1.7%	0.9%	0.4%	0.2%
Number of treated								
2016	720	179	622	916	486	966	8000	450
2017	1325	342	620	928	1173	998	11,700	350
2018	1230	440	648	2446	1632	1164	7100	400
2019	934	468	1360	1267	2806	1816	8500	400
2020	810	364	2300	896	2823	931	3130	230
2021	800	300	1700	500	1500	990	2550	230

**Table 2:** Effect of COVID-19 on HCV elimination.

	Bulgaria	Croatia	Czech Rep.	Hungary	Latvia	Lithuania	Poland	Slovakia
obstruction of access to HCV service	x	x		x	x	x	x	x
obstruction of access to anti-HCV drugs								x
transfer of funds from HCV to COVID-19								
redeployment of staff to care for COVID-19	x	x	x	x		x	x	x
reducing the number of screening tests	x		x	x	x	x	x	

**Table 3:** Availability of national screening programs in particular countries.

	State of Preparation	Comments (Population it Concerns, or Reasons for Unavailability)
Bulgaria	in preparation	applies to the general population
Croatia	not available	insufficient support from health authority
Czech Rep.	in preparation	applies to the general population
Hungary	in preparation	applies to subpopulations
Latvia	not available	lack of political will and financing
Lithuania	in preparation	applies to the general population
Poland	not available	lack of political will and financing
Slovakia	in preparation	applies to subpopulations

**Table 4:** Availability of national screening programs in prisons in particular countries.

	Program Availability	Testing Offered at the Entry (%)	Treatment Offered if Diagnosed (%)	Comments
Bulgaria	in preparation	>90%	no data	no will on the government level, insufficient financing
Croatia	in preparation	occasional	no data	insufficient financing and medical staff
Czech Rep.	ongoing	>90%	90%	_
Hungary	on hold	>90%	50–60%	on hold due to COVID-19, to resume in 2022
Latvia	not available	50–90%	90%	no will on the government and prisoners level
Lithuania	not available	<10%	95–100%	no will on the ministry level, insuff. financing and med. staff
Poland	in preparation	<10%	9%	no will on the government and prison level, insuff. financing
Slovakia	in preparation	10–50%	11%	no will on the ministry level, insuff. financing and med. staff

## Data Availability

Access to data available from the corresponding author.
